# Isolation and Identification of Antioxidative Peptide from Goose Liver Hydrolysate to Ameliorate Alcohol-Mediated Oxidative Stress Damage in HHL-5 Hepatocytes

**DOI:** 10.3390/molecules27217151

**Published:** 2022-10-22

**Authors:** Yeye Du, Zhihong Chen, Haiyang Wei, Shuangjie Zhu, Kezhou Cai

**Affiliations:** 1 School of Biological and Food Engineering, Chuzhou University, Chuzhou 239001, China; 2 Engineering Research Center of Bio-Process, Ministry of Education, Hefei University of Technology, Hefei 230009, China

**Keywords:** goose liver hydrolysate, oligopeptide, hepatocyte protective activity, antioxidant, digestive stability

## Abstract

The aim of this study was to isolate and identify antioxidative peptide from goose liver hydrolysate (GLHP) for ameliorating oxidative stress damage by alcohol in HHL-5 hepatocytes. In this research, the target antioxidative peptides in GLHP were separated, purified, and identified via a tangential flow ultrafiltration system combined with size exclusion chromatography (SEC), ion exchange chromatography (IEC), reversed-phase liquid chromatography (RP-LC), and LC-MS/MS. The results suggested that the amino acid sequence of the target antioxidative peptide for ameliorating alcohol-mediated oxidative stress damage in HHL-5 hepatocytes was Leu-Pro-Leu-Pro-Phe-Pro (LPLPFP), which had a molecular weight of 683.41 Da, and was derived from NADH-ubiquinone oxidoreductase chain 1 in goose liver. In addition, LPLPFP was confirmed to have a satisfactory stability and maintained high hepatic protective activity in a simulated gastrointestinal digestion. Moreover, the mechanism of LPLPFP prevented against oxidative stress damage in HHL-5 hepatocytes was attributed to inhibiting the production of reactive oxide species (ROS) by upregulating genes expression in the *Ahr-NQO1* signal pathway. In conclusion, these results indicated that dietary GLHP supplementation could ameliorate alcohol-mediated oxidative stress damage and provide an affordable dietary intervention strategy to prevent alcohol-mediated hepatocyte damage.

## 1. Introduction

Alcohol-mediated liver damage (ALD) is a primary accountability for morbidity and mortality in worldwide, which was the pathological characteristic, including a simple lipid accumulation in the liver, to serious liver damage such as hepatocyte apoptosis, steatohepatitis, cirrhosis, and hepatocellular carcinoma [1]. ALD is the most common cause of chronic liver disease, and about 2.5 million people die from ALD annually, accounting for 5.9% of global mortality [2]. Despite the efforts of researchers on alcohol-mediated liver damage, the mechanism of its pathology remains unclear. However, recent evidence suggests that reducing oxidative stress damage is a key factor for treating ALD [3]. Oxidative stress is generally regarded as a state of imbalance between reactive oxygen species (ROS) and the antioxidant enzyme system (mainly contains superoxide dismutase, SOD; glutathione peroxidase, GSH-Px; and catalase, CAT) [4].

Mitochondria (via the respiratory chain), endoplasmic reticulum (via CYP2E1), and Kupfer cells (via NADPH oxidase) generate great amounts of various ROS during the ethanol metabolism, mainly hydroxyethyl radical (CH_3_CHOH), superoxide anions (O_2_^−^), and hydroxyl radicals (∙OH), which are the main cause of a lipid peroxidation of the hepatocyte membrane [5]. In addition, hypoxia, bacterial translocation, and the release of proinflammatory cytokines exacerbate the accumulation of ROS, ultimately creating the condition known as oxidative stress. In mammals, GSH-Px, CAT, SOD, and haem oxygenase-1 (HO-1) are essential antioxidant enzymes that eliminate ROS in the body [6]. With a continuous alcohol intake, GSH-Px depletion is contributed to make hepatocytes more sensitive to ROS [7]. In addition, HO-1, as a protective factor against oxidative stress in ALD, plays an anti-oxidative stress role mainly by suppressing the activity of CYP2E1 to reduce the generation of ROS [8]. Consequently, multiple signaling pathways were upregulated by triggering the antioxidant defense system; a large number of intracellular antioxidant enzymes were stimulated to prevent antioxidant damage.

Currently, food-derived bioactive peptides have no obvious adverse effects compared with synthetic drugs (such as metadoxine, amoxicillin clavulanate, rifaximin, and ciprofloxacin), which are attracting more and more interest as alternatives to managing ALD [9]. Recently, various bioactive peptides, such as chicken breast-derived peptide [10], Xuanwei ham peptide [3], and Jinhua ham peptide [11], had been confirmed to have the biological activity of preventing alcohol-mediated liver damage in vivo and in vitro. However, due to price factors, these bioactive peptides are difficult to widely produce and apply. Therefore, the prevention and treatment of ALD remains a huge challenge.

Animal by-products are commonly regarded as a low-cost source of protein for the preparation of bioactive peptides by hydrolysation [12]. In China, approximately 800 million geese are bred annually, accounting for 40% of the world’s total, and a large amount of goose liver is produced as a by-product, which is regarded as a fertilizer or animal feed [13]. Therefore, it is feasible and meaningful to use goose liver to produce bioactive peptides with a hepatocyte-protective activity through a specific enzymatic hydrolysis process.

The aim of this study was to investigate the effects of peptides from goose liver hydrolysate (GLHP) on alcohol-mediated hepatocyte oxidative stress damage and elucidate the mechanisms by which target antioxidant peptides prevent the alcohol-mediated hepatocyte oxidative stress damage. This study may provide an affordable strategy to support research on dietary strategies to prevent alcohol-mediated liver oxidative stress damage.

## 2. Results and Discussion

### 2.1. GLHP Ameliorated Alcohol-Mediated Oxidative Stress Damage in HHL-5 Hepatocytes 

**Figure 1:**
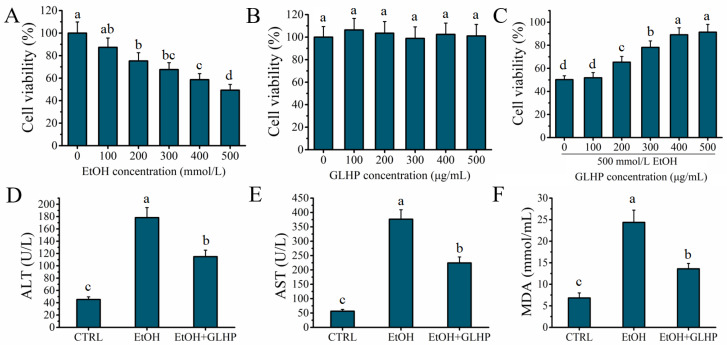
(**A**) Effects of alcohol concentration on HHL-5 hepatocytes viability; (**B**) effects of GLHP concentration on HHL-5 hepatocytes viability; (**C**) effects of the GLHP pre-treatment on HHL-5 hepatocytes viability in an alcohol-mediated HHL-5 hepatocytes injury model (alcohol concentration: 500 mmol/L, GLHP concentration: 400 μg/mL); (**D**) effects of the GLHP pre-treatment on the levels of ALT on model of alcohol-mediated HHL-5 hepatocytes damage; (**E**) effects of the GLHP pre-treatment on the levels of AST on model of alcohol-mediated HHL-5 hepatocytes damage; (**F**) effects of the GLHP pre-treatment on the levels of MDA on model of alcohol-mediated HHL-5 hepatocytes damage; samples with different lower cases letters (a, b, c, and d) were significantly different (*p* < 0.05) when compared to different treatment group.

The cytotoxicity of alcohol and GLHP to HHL-5 hepatocytes was investigated to determine the median lethal dose of alcohol and the treatment concentration of GLHP for use in further experiments. As depicted in Figure 1A, the viability of HHL-5 hepatocytes showed an obvious downward trend with an increasing alcohol treatment concentration (*p* < 0.05). The viability of HHL-5 hepatocytes was approximately 49.28 ± 5.12% at the presence of 500 mmol/L alcohol, which proved that 500 mmol/L was the median lethal dose of alcohol for HHL-5 hepatocytes (LD50). Simultaneously, the viability of HHL-5 hepatocytes did not decrease significantly (*p* > 0.05) with the increase in the GLHP treatment concentration, which confirmed that GLHP was not cytotoxic to HHL-5 hepatocytes (Figure 1B). As shown in Figure 1C, with increasing concentrations of GLHP pre-treatment, the viability of HHL-5 hepatocytes increased significantly (*p* < 0.05) in EtOH + GLHP group. However, the viability of HHL-5 hepatocytes was not significantly (*p* > 0.05) different between 400 and 500 μg/mL of GLHP as the pre-treatment. Consequently, 500 mmol/L and 400 μg/mL were selected as the concentrations of alcohol and GLHP, respectively, for the subsequent experiments. The levels of ALT, AST, and MDA were commonly used as major biomarkers of liver injury after alcohol stimulation [3]. In this study, the indicators (ALT, AST, and MDA) were detected for reflecting the effect of GLHP on alcohol-mediated oxidative stress damage in HHL-5 hepatocytes. As shown in Figure 1D–F, the levels of ALT (l78.29 ± 16.35 U/L), AST (376.57 ± 32.83 U/L), and MDA (24.36 ± 2.85 mmol/mL) in the EtOH group were significantly increased (*p* < 0.05) after the alcohol (500 mmol/L) administration compared with those in the CTRL group. However, the levels of ALT (114.83 ± 10.64 U/L), AST (224.35 ± 20.81 U/L), and MDA (13.59 ± 1.26 mmol/mL) in the EtOH + GLHP group were significantly decreased (*p* < 0.05) when compared to the EtOH group. These results confirmed that a pre-treatment with GLHP could ameliorate the hepatocytes oxidative stress damage mediated by alcohol.

### 2.2. GLHP Was Separated by Size Exclusion Chromatography (SEC)

**Figure 2:**
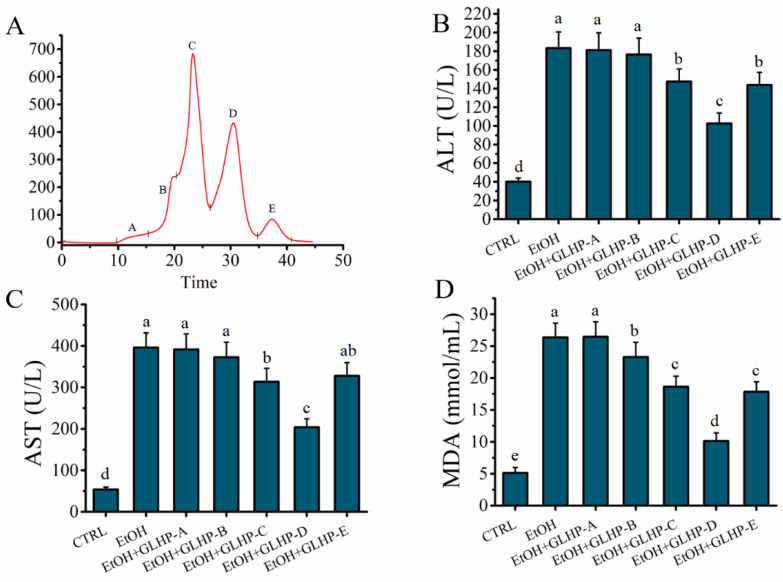
Effects of peptides obtained by size exclusion chromatography on model of alcohol-mediated HHL-5 hepatocytes damage. (**A**) Sephadex^TM^ 10/300 GL size exclusion chromatography of peptides from GLHP; (**B**–**D**): effects of GLHP-(A–E) pre-treatment on levels of ALT, AST, and MDA on model of alcohol-mediated HHL-5 hepatocytes damage. Samples designated with different lower cases letters (a, b, c, d, and e) were significantly different (*p* < 0.05) when compared to different treatment group.

GLHP could ameliorate alcohol-induced hepatocytes oxidative stress damage. However, GLHP is a collection of many oligopeptides with molecular weights less than 3 kDa. Elucidating the characteristics of the target antioxidant oligopeptide is a question worthy of further investigation. In this study, these mixtures of GLHP were separated according to the different molecular masses used by SEC. As shown in Figure 2A, the mixture of GLHP was divided into five components, A to E, with A representing the maximum molecular mass and the E representing the minimum. The hepatocyte protective activity of the fraction (A–E) obtained by SEC was evaluated in alcohol-mediated HHL-5 hepatocytes in vitro. The results show that the levels of ALT (102.59 ± 11.26 U/L), AST (203.73 ± 20.54 U/L), and MDA (10.15 ± 1.27 mmol/mL) in the fraction D group were significantly lower (*p* < 0.05) than those in the other fraction groups, which confirmed that fraction D contained the target antioxidant oligopeptide for ameliorating the hepatocytes oxidative stress damage associated with alcohol. Therefore, the fraction D was stored for use after being freeze-dried.

### 2.3. Fraction D of GLHP Was Separated by Ion Exchange Chromatography (IEC)

**Figure 3:**
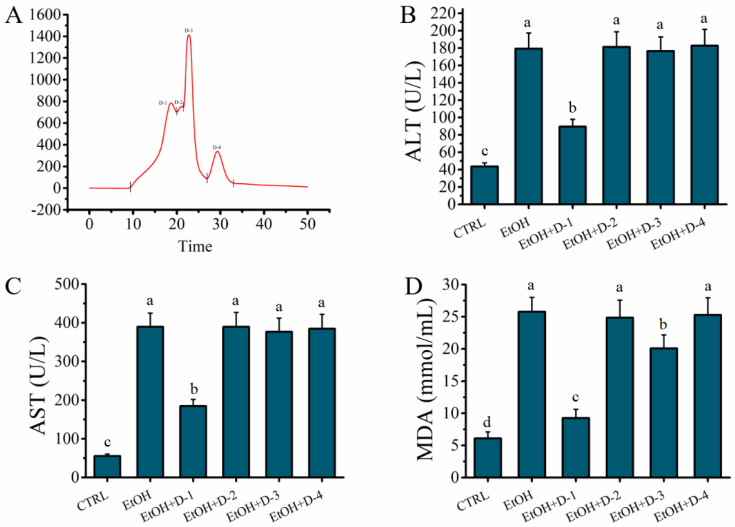
Effects of peptides obtained by ion exchange chromatography on model of alcohol-mediated HHL-5 hepatocytes damage. (**A**) HiTrap Capto DEAE ion exchange chromatography of peptides from GLHP-D; (**B**–**D**): effects of GLHP-D (1–4) pre-treatment on levels of ALT, AST, and MDA on model of alcohol-mediated HHL-5 hepatocytes damage. Samples designated with different lower cases letters (a, b, c, and d) were significantly different (*p* < 0.05) when compared to different treatment group.

Fraction D represented a mixture of peptides with similar small molecular masses. However, the peptide fragments in fraction D may contain groups with different anion exchange capacities. Therefore, fraction D was further separated by IEC depending on the different anion exchange capacities to further elucidate the characteristics of the target peptide. As shown in Figure 3A, the mixture of fraction D was separated into four fractions (D-1, D-2, D-3, and D-4); the D-1 was the relatively weaker anion exchange capacity, while the D-4 was the stronger. The hepatocyte protective activity of the fractions obtained by IEC was evaluated in an alcohol-mediated HHL-5 hepatocytes in vitro. As shown in Figure 3B–D, the levels of ALT (89.54 ± 8.37 U/L), AST (184.55 ± 17.26 U/L), and MDA (9.25 ± 1.37 mmol/mL) in the fraction D-1 group were significantly lower (*p* < 0.05) than those in the other fraction groups, which confirmed that fraction D-1 contained the target antioxidant oligopeptide for ameliorating the hepatocytes oxidative stress damage associated with alcohol. Finally, fraction D-1 was stored for use after being freeze-dried.

### 2.4. D-1 Was Separated by RP-LC

**Figure 4:**
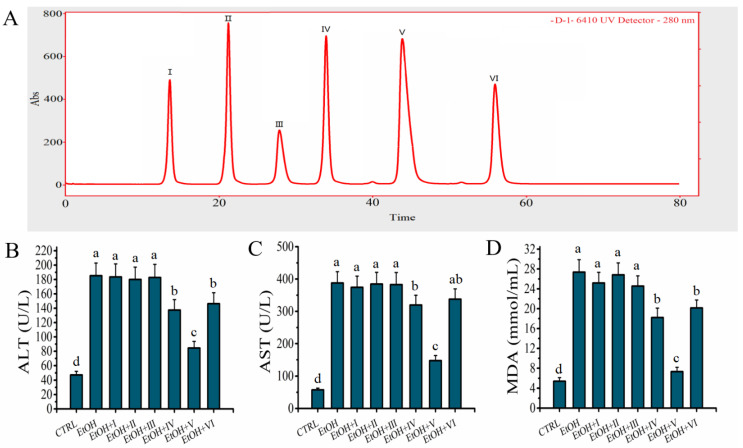
Effects of peptides obtained by reversed-phase liquid chromatography on model of alcohol-mediated HHL-5 hepatocytes damage. (**A**) BEH C18 reversed-phase liquid chromatography of peptides from D-1; (**B**–**D**): effects of D-1 (I–VI) pre-treatment on levels of ALT, AST, and MDA on model of alcohol-mediated HHL-5 hepatocytes damage. Samples designated with different lower cases letters (a, b, c, and d) were significantly different (*p* < 0.05) when compared to different treatment group.

Fraction D-1 represented a mixture of peptides with similar small molecular masses and lower anion exchange capacities. However, these peptide fragments in fraction D-1 may contain components with differences in hydrophobicity. Therefore, fraction D-1 was further separated by RP-LC depending on the differences in hydrophobicity to further elucidate the characteristics of the target peptide. As shown in Figure 4A, the collection of fraction D-1 was separated into six fractions (Ⅰ, Ⅱ, Ⅲ, Ⅳ, Ⅴ, and Ⅵ); the Ⅵ represented the strongest hydrophobicity, while the Ⅰ represented the weakest. The antioxidant activity of fraction Ⅰ–Ⅵ obtained by RP-LC was evaluated in an alcohol-mediated HHL-5 hepatocytes in vitro. Compared to the other fractions, the levels of ALT (84.51 ± 9.27 U/L), AST (147.27 ± 15.89 U/L), and MDA (7.32 ± 1.08 mmol/mL) were significantly lower in the group which received a pre-treatment with fraction 5 (Figure 4B–D), which confirmed that fraction 5 showed the strongest hepatocyte protective activity. Finally, fraction 5 was collected and freeze-dried for further analysis.

### 2.5. The Target Peptide Was Determined by LC-MS/MS

**Figure 5:**
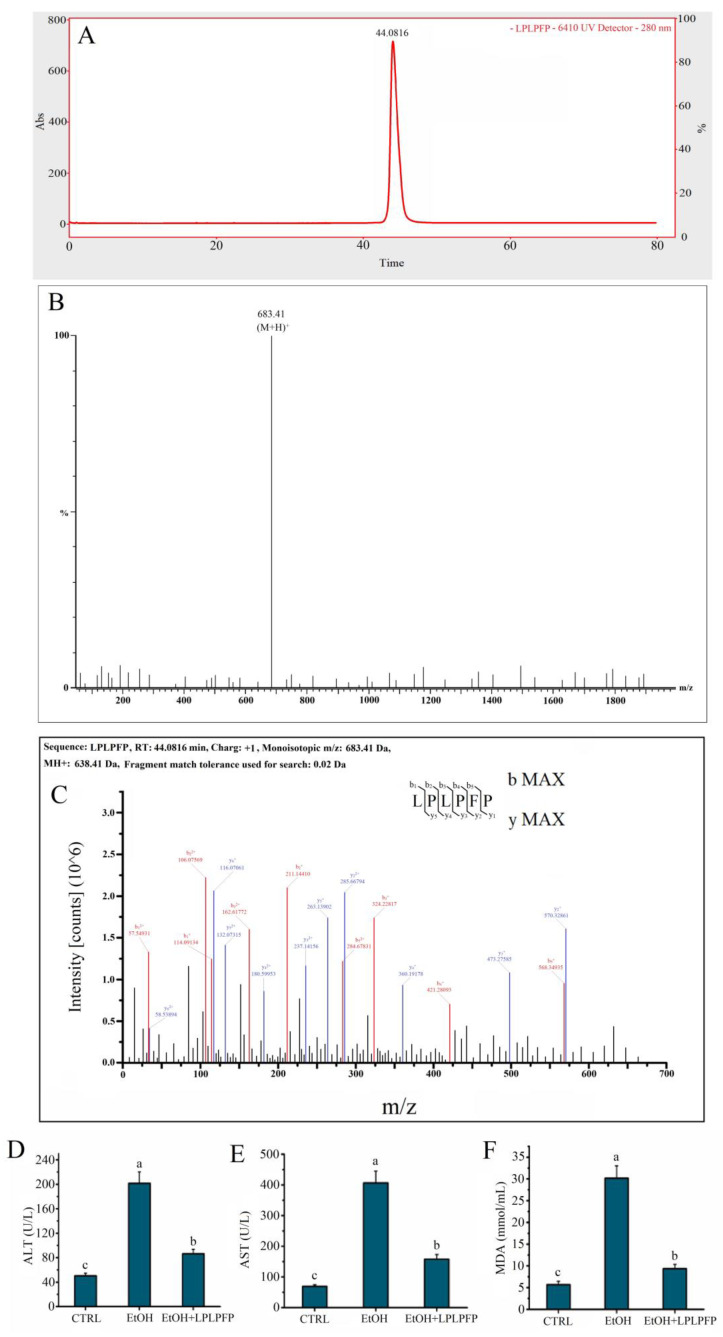
(**A**) The total particles of D-1-V in MS/MS spectrum; (**B**) mass spectrum of peak at 44.0816 min; (**C**) identification of the molecular weight and amino acid sequence of the purified D-1-V by MS/MS spectrum; (**D**–**F**): effects of solid phase synthesis of LPLPFP pre-treatment on levels of ALT, AST, and MDA on model of alcohol-mediated HHL-5 hepatocytes damage. Samples designated with different lower cases letters (a, b, and c) were significantly different (*p* < 0.05) when compared to different treatment group.

According to the obtained results, the target GLHPs were characterized as having low molecular weights, high hydrophobicity, and weak anion exchange capacities. In this study, only one major peak is shown in Figure 5A, which suggested one main target peptide in fraction 5. As shown in Figure 5B, the molecular ion peak of the target antioxidant oligopeptide was detected at m/z 683.41, which indicated that the molecular weight of the target antioxidant oligopeptide was 683.41 Da. Simultaneously, the primary structure of the target antioxidant oligopeptide was Leu-Pro-Leu-Pro-Phe-Pro (LPLPFP), which was derived from the NADH-ubiquinone oxidoreductase chain 1 in goose liver (Figure 5C). The content of LPLPFP in the hydrolysates of goose liver was about 3.83%. Some characteristics of LPLPFP are a low molecular weight (683.41 Da), a high hydrophobicity (a large amount of hydrophobic amino acid residues, such as Leu, Pro, and Phe), and a weak anion exchange capacity (charge: +1), which conformed to the identified characteristics. 

In this study, LPLPFP was synthesized by chemical solid-phase synthesis to further verify the HHL-5 hepatocytes protective activity of the target antioxidant oligopeptide on the oxidative stress damage associated with alcohol in vitro. The results shown that a pre-treatment with the target antioxidant oligopeptide synthesized in the solid phase significantly ameliorated the HHL-5 hepatocytes oxidative stress damage associated with alcohol in vitro (Figure 5D–F), which further confirmed that LPLPFP isolated from enzymatic hydrolysates of goose liver ameliorated the HHL-5 hepatocytes oxidative stress damage associated with alcohol.

### 2.6. The Antioxidant Activity of LPLPFP

In addition, the protective function of LPLPFP against the oxidative stress damage mediated by alcohol in hepatocytes is connected with the oxidation resistance. Typically, oligopeptides containing 2 to 10 amino acid residues are considered to have a higher antioxidant activity compared with their parent protein [14]. Free radicals, linked to oxidative stress, are highly reactive with other molecules, including vital DNA and proteins, the destruction of which can damage or kill cells. Among the many free radicals, the ∙OH radical is commonly considered as one of the most harmful highly reactive molecules for attacking and damaging cellular proteins, lipids, and DNA [15]. Therefore, the levels of the DPPH, ∙OH radical scavenging activity, and Fe^2+^ chelation capacity were commonly considered as major indexes for evaluating the oxidation resistance of peptides [16]. In this study, the results show that the level of the DPPH radical scavenging rate of LPLPFP (1 mg/mL) was approximately 95.74 ± 2.86%, whereas BHT showed about 90.56 ± 1.73% at the same concentration. This result confirmed that the DPPH radical scavenging rate of LPLPFP was higher than that of tuna dark muscle hydrolysate (the scavenging rate of DPPH: 31.5%, 3 mg/mL) [17] and rice protein hydrolysate (the scavenging rate of DPPH: 44.31%, 1.5 mg/mL) [18]. This may be attributed to the large number of hydrophobic amino acid residues contained in LPLPFP, such as Leu, Pro, and Phe [19]. In addition, the level of the ∙OH radical scavenging rate of LPLPFP (1 mg/mL) was approximately 88.45 ± 1.52%, whereas BHT showed about 79.38 ± 2.03% at the same concentration (Table 1). This value is higher than that of various other protein hydrolysates, such as bullfrog skin (the scavenging rate of ∙OH: 47.6%, 1.5 mg/mL) [20] and marine *chlorella ellipsoidea* hydrolysate (the scavenging rate of ∙OH: 50.0%, 2.698 mg/mL) [21]. Meanwhile, the Fe^2+^ chelating ability of LPLPFP (35.32 ± 4.01%) was higher than that of the BHT (14.39 ± 2.46%), but lower than that of the Gly-Lys-Phe-Asn-Val from Jinhua ham (the scavenging rate of Fe^2+^: 63.20%, 1 mg/mL) [16] and Asn-Pro-Pro-Lys-Phe-Asp from Xuanwei ham (the scavenging rate of Fe^2+^: 66.92%, 1 mg/mL) [3]. The low level of the Fe^2+^ chelating ability of LPLPFP was associated with the lack of acid or basic amino acid residues in the target peptide [18].

### 2.7. Effects of Pepsin-Trypsin Simulated GI Digestion on the Stability of LPLPFP

**Figure 6:**
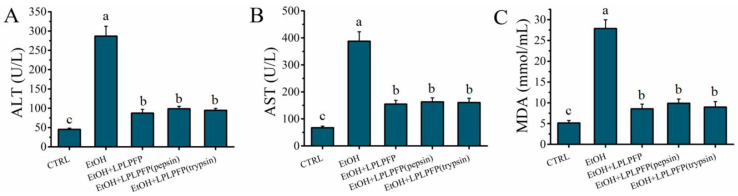
(**A**–**C**) Effects of the LPLPFP pre-treatment with pepsin/trypsin-simulated gastrointestinal digestion on the levels of ALT, AST, and MDA on model of alcohol-mediated HHL-5 hepatocytes damage; different letters (a, b, and c) in figures indicate significant differences (*p* < 0.05).

The structure and function of the peptides was mainly affected by the GI digestion. In this study, the stability of LPLPFP during the GI digestion was evaluated in a pepsin-trypsin simulated GI digestion model in vitro. As shown in Figure 6A–C, the levels of ALT (98.57 ± 6.32 U/L), AST (163.15 ± 15.32 U/L), and MDA (8.95 ± 1.34 mmol/mL) after digestion with pepsin for 2.0 h were slightly increased compared to the levels of ALT (87.36 ± 9.75 U/L), AST (154.83 ± 14.26 U/L), and MDA (8.54 ± 1.15 mmol/mL) in the EtOH + LPLPFP group, but these levels were not significantly difference (*p* > 0.05). Furthermore, the levels of ALT (94.46 ± 5.26 U/L), AST (160.89 ± 16.04 U/L), and MDA (8.95 ± 1.34 mmol/mL) were not significantly increased (*p* > 0.05) after digestion with trypsin for 2.0 h compared to that in the EtOH + LPLPFP and EtOH + LPLPFP (pepsin) groups. These results indicated that LPLPFP had a strong resistance to pepsin-trypsin simulated GI digestion. In summary, LPLPFP has a satisfactory stability and resistance to GI digestion.

### 2.8. The Mechanism of LPLPFP Ameliorated Alcohol-Mediated Oxidative Stress in HHL-5 Hepatocytes

**Figure 7:**
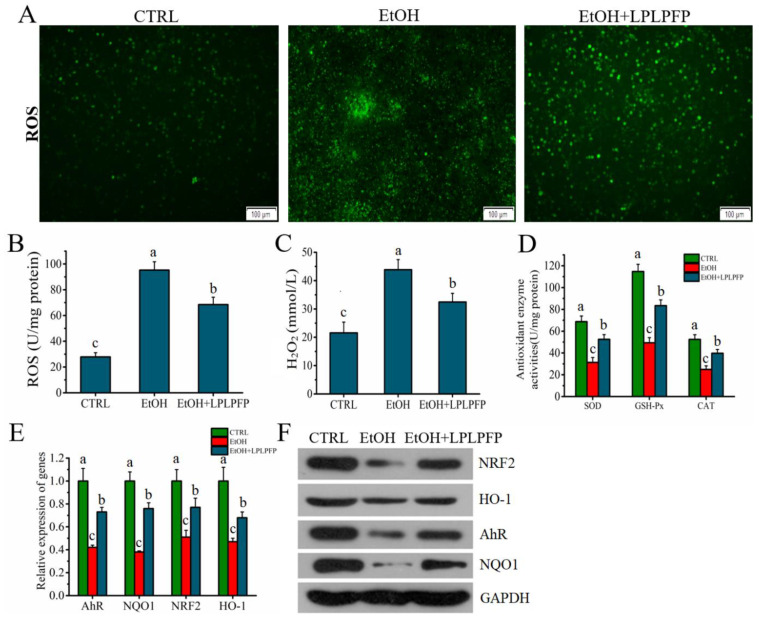
(**A**,**B**) Effects of LPLPFP on the levels of ROS in alcohol-mediated HHL-5 hepatocytes; (**C**) effects of LPLPFP on the levels of H_2_O_2_ in alcohol-mediated HHL-5 hepatocytes; (**D**) effects of LPLPFP on the levels of antioxidant enzyme activity in alcohol-mediated HHL-5 hepatocytes; (**E**) effects of LPLPFP on the mRNA expression levels of *AhR*, *NQO1*, *HO-1,* and *NRF2* in alcohol-mediated HHL-5 hepatocytes; (**F**) effects of LPLPFP on the protein expression levels of *AhR*, *NQO1*, *HO-1,* and *NRF2* in alcohol-mediated HHL-5 hepatocytes; samples designated with different lower cases letters (a, b, and c) were significantly different (*p* < 0.05) when compared to different treatment group.

Oxidative stress was commonly considered as a crucial factor for hepatocytes damage associated with alcohol [22]. HHL-5 hepatocytes generate excess ROS and H_2_O_2_ in response to alcohol treatment, which accelerates hepatocytes damage through oxidative stress [11]. As shown in Figure 7A–C, stimulation with alcohol significantly increased the levels of the ROS (95.23 ± 6.54 U/mg prot) and H_2_O_2_ (43.86 ± 3.57 mmol/L), but the levels of the ROS (68.47 ± 5.61 U/mg prot) and H_2_O_2_ (32.45 ± 3.06 mmol/L) were significantly (*p* < 0.05) decreased after a pre-treatment with LPLPFP. The excess ROS and H_2_O_2_ in hepatocytes were scavenged by antioxidant enzymes SOD, GSH-Px, and CAT for resisting ROS -mediated oxidative stress [6]. As shown in Figure 7D, the levels of SOD (31.28 ± 4.53 U/mg prot), GSH-Px (49.26 ± 4.82 U/mg prot), and CAT (24.87 ± 3.28 U/mg prot) were significantly (*p* < 0.05) decreased after the administration of alcohol than that of the CTRL group. However, the decrease trends of SOD (52.46 ± 4.37 U/mg prot), GSH-Px (83.38 ± 5.26 U/mg prot), and CAT (39.72 ± 3.47 U/mg protein) mediated by alcohol were significantly decelerated by a pre-treatment with LPLPFP. (Figure 7D). The recent evidence suggested that the aryl hydrocarbon receptor (AhR) played a crucial role in the oxidative defense system by activating the NRF2 signaling pathway, which contributed to the antioxidant response by regulating multiple antioxidant enzymes, such as SOD, GSH-Px, CAT, NQO1, and HO-1 [23,24]. As shown in Figure 7E,F, the mRNA and protein expression levels of *AhR*, *NQO1*, *NRF2,* and *HO-1* were significantly decreased (*p* < 0.05) in the HHL-5 cells stimulated with alcohol, but the decrease trends of the mRNA and protein expression levels of *AhR*, *NQO1*, *NRF2,* and *HO-1* mediated by alcohol were significantly decelerated by a pre-treatment with LPLPFP, which confirmed that LPLPFP could enhance the antioxidant defense of cells to reduce ROS accumulation and ameliorate the alcohol-mediated oxidative stress damage response by upregulating the *AhR-NQO1* signaling pathway. In addition, we hypothesized that LPLPFP could activate the *AhR-NQO1* signaling pathway to resist alcohol-mediated oxidative stress damage in HHL-5 hepatocytes, which may be related to the Phe residues contained in it. However, we are investigating this part of the work and will not discuss it further here.

## 3. Materials and Methods

### 3.1. Preparation of GLHP

The endogenous enzymes of goose liver were inactivated by crushing and heating at 95 °C for 5 min. Then, the goose liver was vacuum freeze-dried to form a powder. One hundred grams of goose liver powder was accurately weighed and placed in a beaker, 2000 mL of PBS solution (0.2 mmol/L, pH 7.2) was added and homogenized for 40 s (4 strokes, 10 s per stroke) with the polytron homogenizer (IKA T25 digital ultra-turrax, IKA, Germany) at a speed of 22,000 rpm on ice. The preparation of the goose liver hydrolysate was carried out by pepsin enzymatic hydrolysis, which was slightly modified according to the study by Chou et al. [25]. A total of 250 mg of pepsin enzymes (3000 U/mg) were accurately weighed and added to the goose liver homogenate and the pH was 3.0 (adjusted with 6 mol/L HCl solution) for hydrolysis at 37 °C for 4 h. The supernatant was isolated from the hydrolysate by centrifugation at 4 °C 12,000× *g* for 20 min and filtrated by tangential flow filtration (TFF) (P/N: S02-E003-05-N, media/rating: mPES/3 kDa, surface area: 790 cm^2^); peptides smaller than 3 kDa after ultrafiltration were reserved for use. Then, the dialysis solution was desalted by a C18-SPE column (Agela Technologies Inc., Tianjin, China) with 80% methanol. The mixture was collected and freeze-dried for subsequent experiments.

### 3.2. Isolation and Identification of Bioactive Peptides from GLHP

The methods for the isolation and identification of bioactive peptides from GLHP were based on the description reported by Nie et al. [3]. Primarily, the 100 mg/mL of GLHP was separated into different fractions depending on the different molecular masses by the size exclusion chromatography (SEC) configured with a Sephadex column (Sephadex^TM^ 10/300 GL, 10 × 300 mm, General Electric Company, Marlborough, MA, USA). The eluent was a hydrochloric acid solution of 0.1 mol/L at a flow rate of 1 mL/min. The fractions were detected at 280 nm by an ultraviolet detector and collected by an automatic fraction collector. 

Then, the fraction of peptide with the highest hepatocyte protective activity obtained from SEC was further separated into different fractions depending on the different anion exchange capacities. A total of 100 mg/mL of peptide was separated by ion exchange chromatography (IEC) configured with an ion exchange column (HiTrap Capto DEAE, 5 mL, 1.6 × 2.5 cm, General Electric Company, Marlborough, MA, USA). Twenty mM of Tris-HCl (pH 8.0) was used as a starting buffer, and 20 mM of Tris-HCl/1 M NaCl (pH 8.0) was used as an elution buffer. The elution program: (1) wash with 5 column volumes of starting buffer; (2) elute with a gradient volume of 10 column and an increasing salt concentration up to 0.5 M of NaCl (50% elution buffer); and (3) wash with 5 column volumes of 1 M of NaCl (100% elution buffer). The above flow rates were 5 mL/min. The fractions were detected at 280 nm by an ultraviolet detector and collected by an automatic fraction collector. 

Moreover, the fraction of peptide with the highest hepatocyte protective activity obtained from the IEC was further separated into different fractions depending on the different hydrophobicity. Ten mg/mL of peptide was separated by reversed-phase liquid chromatography (RP-LC) configured with a reversed phase column (1.7 μm, 2.1 × 100 mm, Waters Technology Co., Ltd, Shanghai, China). Eluent A: 0.065% TFA in 2% acetonitrile; eluent B: 0.050% TFA in 80% acetonitrile. The flow gradient was set as follows: 0–30 min, 100% eluent A; 30–60 min, 30–80% eluent B; 60–80 min, 100% eluent A. The above flow rates were 0.5 mL/min. The fractions of peptide were detected at 280 nm by an ultraviolet detector and collected by an automatic fraction collector. 

The fraction of peptide with the highest hepatocyte protective activity obtained from RP-LC was further identified by LC-MS/MS configured with a reversed phase BEH C18 analytical column (1.7 μm, 2.1 × 100 mm, Waters Inc.). The mobile phase and gradient elution procedures were performed in the same manner as above. The fractions were detected at 280 nm by an ultraviolet detector. The flow entered directly into the MS/MS system for a multiple reaction measurement. The instrumental operation and mass spectrogram information analysis were used by Mass Lynx V4.1.

### 3.3. Measurement of Antioxidant Activity

The antioxidant activity of the target peptides was evaluated by detecting the hydroxyl radical scavenging activity (∙OH), DPPH radical scavenging activity, and Fe^2+^ chelating ability; the method was performed according to Zhu et al. [16] with some modifications. 

Measurement of the ∙OH radical scavenging activity. The sample group was a mixture of 0.6 mL of 1, 10-phenanthroline monohydrate (5 mmol/L) and 0.4 mL of phosphate buffer (0.2 M, pH 7.4), and then 0.6 mL of LPLPFP (1 mg/mL), 0.6 mL of EDTA (15 mmol/L), and 0.6 mL of FeSO4 (5 mmol/L) were mixed thoroughly. After mixing, 0.8 mL of H_2_O_2_ (0.1%) was added and incubated at 37 °C for 60 min. Afterward, the mixture absorbance was measured at 536 nm with the multifunctional microplate reader. The control group contained the same solutions as the sample group, except deionized water was used instead of the sample. The blank group contained the same solution as the control group, except deionized water was used instead of H_2_O_2_. The results were determined according to the equation.

*hydroxyl radical scavenging activity* (%) = (*As* − *Ac*) ÷ (*Ab* − *Ac*) × 100%

where *As*, *Ac*, and *Ab* represent the absorbance of the sample and the control and blank groups, respectively. BHT was used as a positive control.

Measurement of the DPPH radical scavenging activity. One mL of the LPLPFP (dissolved in deionized water) was mixed with 1 mL of 0.2 mmol/L DPPH (in 95% ethanol) as the sample group; 1 mL of deionized water was mixed with 1 mL of ethanol (95%) as the blank group; and 1 mL of 0.2 mmol/L DPPH was mixed with 1 mL of ethanol (95%) as the control group. The mixture was agitated using a vortex and incubated for 30 min at room temperature, protected from light, and then the absorbance at 517 nm was measured. The scavenging activity of LPLPFP and BHT was calculated using the following equation:

*DPPH radical scavenging activity* (%) = 1 − [ (*As* − *Ac*) ÷ (*Ab* − *Ac*)] × 100%

where *As*, *Ac*, and *Ab* represent the absorbance of the sample, the control, and the blank groups, respectively. BHT was used as a positive control.

Measurement of the Fe^2+^ chelating ability. Briefly, 1 mL of LPLPFP was mixed with 0.05 mL of FeCl_2_ (2 mmol/L) and 0.2 mL of ferrozine (5 mmol/L). The mixture was vortexed and kept at room temperature for 10 min prior to the measurement of the absorbance at 562 nm (*As*). The control (*Ac*) contained everything except using deionized water instead of LPLPFP. The chelating ability was calculated according to the equation

*Fe*^2+^*chelating ability* (%) = (*Ac* − *As*) ÷ *Ac* × 100%

where *As* and *Ac* represent the absorbance of the sample and the control groups. BHT was used as a positive control.

### 3.4. Cell Culture and Treatments

HHL-5 human embryonic hepatocytes were purchased from American Type Culture Collection (ATCC) (University Boulevard Manassas, VA 20110 USA); the cells were cultured and treated according to Nie et al. [3], with some modifications. The cell suspension was inoculated into 96-well plates (4 × 10^3^ per well) and added to the culture medium containing: DMEM-F12 (1:1, HyClone, Logan, UT, USA), 10% FBS (Gibco, New York, NY, USA), 1% P/S (100 U/L), 15 mmol/L of HEPES, and 2.5 mmol/L of L-glutamine, then the plates with cells were placed in a 5% CO_2_ humidified atmosphere at 37 °C for 24 h. After it grew to an approximately 70% confluency, 100, 200, 300, 400, and 500 mmol/L of anhydrous ethanol was added in the EtOH group, respectively, and incubated for 48 h. Then, the cell viability was evaluated by a Cell Counting Kit-8 assay to determine the optimal concentration of alcohol needed to induce HHL-5 hepatocytes damage. Furthermore, HHL-5 hepatocytes were cultured with different concentrations of GLHP (100 μg/mL, 200 μg/mL, 300 μg/mL, 400 μg/mL, and 500 μg/mL) to evaluate the cytotoxicity of GLHP, as described above. The ability of GLHP to protect HHL-5 hepatocytes was evaluated. In the EtOH + GLHP group, 100, 200, 300, 400, and 500 μg/mL of GLHP was added, respectively, and treated for 12 h, then replaced with the culture medium containing 500 mmol/L of anhydrous ethanol, and cultured for 48 h. In the EtOH group, a culture medium supplemented with an equal volume of PBS was added and incubated for 12 h, and the culture medium was then substituted with 500 mmol/L of anhydrous ethanol and incubated for 48 h. In the CTRL group, a culture medium supplemented with an equal volume of the PBS was added and incubated for 12 h, and the culture medium was then substituted with an equal volume of the PBS and incubated for 48 h. Finally, the culture media and cells were collected for further analysis.

### 3.5. Simulated Gastrointestinal (GI) Digestion

The stability of LPLPFP in response to pepsin and trypsin in the simulation of GI digestion in vitro was examined based on Xie et al. [26]. A 1 L ultrapure water solution containing 2 g of NaCl and 3.2 g of pepsin (3000 U/mg protein) with a pH of 3.0 (adjusted with a 6 mol/L HCl solution), was configured as simulated gastric juice. In addition, 0.68 g of potassium dihydrogen phosphate, 7.7 mL of 0.2 mol/L NaOH solution, 1 g of trypsin (285 U/mg protein), and 6 g of bile salt were added and dissolved in 70 mL of ultrapure water with a pH of 7.6 (adjusted with a 0.2 mol/L NaOH solution) and the volume was filled with ultrapure water up to 1 L (as simulated intestinal fluid). The 400 μg/mL of peptide solution at pH 3.0 (adjusted with a 1 mol/L HCl solution) and 5 mL of simulated gastric juice was mixed and shaken at 37 °C for 2 h to simulate gastric digestion, then the mixture was terminated by being heated at 100 °C for 10 min, the pH was adjusted to 7.2 with a 0.2 mol/L NaOH solution, and 5 mL of simulated intestinal juice was added to the solution, and the same conditions were used for simulating intestinal digestion. Finally, the digestive liquid was transferred to the ultrafiltration centrifuge tube (1 kDa) and centrifuged at 4 °C, 8500× *g* for 20 min; the filtered solutions were desalted by a polar enhanced polymer (PEP) column (500 mg/6 mL) and dried in a vacuum freeze-dryer.

### 3.6. Determination of Cell Viability

The Cell Counting Kit-8 (CCK-8, Beijing Solarbio Science and Technology Co., Ltd., Beijing, China) was used to assess the HH-5 cell viability; the testing procedures were performed according to the manufacturer’s protocol. Adherent cells in 96-well culture plates were treated and then incubated with a CCK solution for 2 h; the absorbance was detected at a wavelength of 450 nm with the multifunctional microplate reader.

### 3.7. Determination of Oxidative Stress Indicators

Cells were collected with PBS, the activity of ALT (C009-2-1), AST (C0010-2-1), and MDA (A003-1) in cell supernatant and the SOD (A001-3), GSH-Px (A005), CAT (A007-1-1), and ROS (E004-1-1) in the cells were measured using corresponding assay kits (Nanjing Jiancheng Biotechnology Institute, Nanjing, China) according to the manufacturer’s instructions.

ALT and AST: 5 μL of sample was mixed with 20μL of ALT/AST matrix solution and the reaction was performed at 37 °C for 30 min. Then, 20 μL of 2, 4-dinitrophenylhydrazine solution was added and the reaction was performed at 37 °C for 20 min. Finally, 200 μL of sodium hydroxide solution (0.4 mol/L) was added and the reaction was performed at room temperature for 15 min. The absorbance value was measured at a 510 nm wavelength by a multifunctional microplate reader.

MDA: 0.1 mL of sample was mixed with 0.1 mL of reagent one, 3 mL of reagent two, and 1 mL of reagent three in the 10 mL centrifuge tube. Then, the centrifuge tube was centrifuged at 4 °C, 3500× *g* for 10 min after being heated in a boiling water bath for 40 min. The absorbance value of 200 μL of the supernatant was measured at a 532 nm wavelength by a multifunctional microplate reader.

SOD: 20 μL of sample was mixed with 20 μL enzyme working liquid and 200 μL of substrate application solution. Then, miscible liquids were performed at 37 °C for 20 min. The absorbance values of the miscible liquids were measured at a 450 nm wavelength by a multifunctional microplate reader.

GSH-Px: 0.2 mL of sample was mixed with 0.2 mL of GSH-Px (1 mmol/L) and performed at 37 °C for 5 min. Then, 0.1 mL of reagent one was added and performed at 37 °C for 5 min. Subsequently, the miscible liquids were centrifuged at 4 °C, 3500× *g* for 10 min. One mL of supernatant was mixed with 1 mL of reagent three, 0.25 mL of reagent four, and 0.05 mL of reagent five and performed at room temperature for 15 min. The absorbance value was measured at a 412 nm wavelength by a multifunctional microplate reader.

CAT: 0.05 mL of sample was mixed with 1.0 mL reagent one, 0.1 mL reagent two and performed at 37 °C for 1 min. Then, 1.0 mL reagent three and 0.1 mL reagent four were mixed with the miscible liquids. The absorbance value was measured at 405 nm wavelength by a multifunctional microplate reader.

ROS: A DCFH-DA probe was added directly to the serum-free medium: DCFH-DA was generally diluted with a serum-free medium at a final concentration of 10 µM at 1:1000. After removing the culture medium, an appropriate volume of diluted DCFH-DA was added. The volume added should be sufficient to cover the cells. Usually, no less than 1 mL of diluted DCFH-DA is added to one well of a 6-well plate. A sample of cells without a probe and with a medium only was set as negative for the control. Positive control: take a sample of cells with a probe and add reactive oxygen species to induce the cells. The cells were incubated at 37 °C for 20 min for several hours (mixed upside down every 5 min to make full contact between the probe and cell). The length of the incubation time was related to the cell type, stimulation condition, and DCFH-DA concentration. In general, an obvious green fluorescence can be observed in the positive control cells after 30 min of stimulation. The culture medium was aspirated, and the serum-free culture medium or 0.01 MPBS was used to blow repeatedly. The bottom of the bottle was observed to turn transparent from translucent (cell monolayer connected into slices), and almost all of the cell layer was blown into the PBS. The cell suspension was completely collected into a 1.5 mL centrifuge tube. The DCFH-DA was washed twice with a serum-free medium or PBS to fully remove the DCFH-DA that did not enter the cells. At 1000 rpm/min for 5 min, the supernatant was aspirated and then the cells were resuspended with PBS for determination. Wavelength setting: the optimal excitation wavelength is 488 nm, and the optimal emission wavelength is 525 nm. It can also be detected according to FITC fluorescence detection conditions. The results were expressed as fluorescence values.

### 3.8. RT-PCR Analysis

The total RNA was extracted from the cells by using Trizol (Invitrogen, Carlsbad, CA) according to the manufacturer’s protocol and the determination of the concentration was done by the Nanodrop 2000 (Thermofisher, Waltham, MA, USA). Subsequently, the extracted RNA was used to reverse cDNA using the high-capacity cDNA Reverse Transcription kit (Applied Biosystems, Foster City, CA, USA). Finally, RT-PCR was performed using a Power UP SYBR green Master Mix (Applied Biosystems, Foster City, CA, USA) on a Heal Force CG-02 thermocycler (Heal Force, Shanghai, China). The primer sequences of the genes were recorded in the Supplementary Materials Table S1, the gene *GAPDH* was set as the internal reference gene. The mRNA expression levels were calculated using the 2^−ΔΔCt^ method.

### 3.9. Western Blot Analysis

The proteins in the HHL-5 hepatocytes were extracted by homogenizing after adding 1 mM of PMSF solution and 200 μL of RIPA buffer, and the proteins present in the supernatant were collected and measured for concentration with a BCA protein assay kit (Nanjing Jiancheng Bioengineering Institute, Nanjing, China). Then, the proteins were fully denatured by adding a 5× reduced gel sample loading buffer (4:1) and heating in a 100 °C bath for 15 min. The target proteins were isolated by SDS–PAGE (concentrating gel voltage: 75 V, separating gel voltage: 120 V) and transferred to polyvinylidene difluoride membranes (Servicebio, Wuhan, China). The membranes were blocked with 5% skimmed milk at room temperature for 30 min. Subsequently, they were incubated with the primary antibodies (NRF2, HO-1, AhR, NQO1, and GAPDH, Abcam plc, Cambridge, UK) at 4 °C in a shaker overnight and the secondary antibody (anti-rabbit and anti-mouse IgG (H + L), CST, Boston, MA, USA) was incubated at room temperature for 30 min, respectively. Finally, the protein bands were visualized by an ECL chemiluminescence kit (Thermo Scientific, Waltham, MA, USA). The bands were quantified and analyzed by ImageJ software (V1.8.0.112).

### 3.10. Statistical Analysis

All experiments were conducted at least in triplicates. All results are expressed as the mean ± SD. The SAS software (version 9.0) was applied to calculate the significance (*p* < 0.05) among the samples by Duncan’s multiple-range test (20) in a one-way analysis of variance (ANOVA).

## 4. Conclusions

In conclusion, the primary structure of the target antioxidant oligopeptide was Leu-Pro-Leu-Pro-Phe-Pro (LPLPFP), which had a molecular weight of 683.41 Da and derived from the NADH-ubiquinone oxidoreductase chain 1 in goose liver. In addition, LPLPFP was confirmed to have satisfactory effects on maintaining a high hepatocyte protective activity after simulated GI digestion. The mechanism of LPLPFP prevented against oxidative stress damage in HHL-5 hepatocytes was attributed to inhibiting the production of ROS by upregulating the genes expression in the *Ahr-NQO1* signal pathway. In summary, this study indicated that dietary GLHP may be an affordable and effective dietary intervention strategy to the prevent oxidative stress damage mediated by alcohol.

## Figures and Tables

**Table 1:** Antioxidant Activity of LPLPFP.

	Content %	Concentration (mg/mL)	DPPH Radical Scavenging Activity (%)	∙OH Radical Scavenging Activity (%)	Fe^2+^ Chelating Ability (%)
LPLPFP	3.83%	1	95.74 ± 2.86 ^a^	88.45 ± 1.52 ^a^	35.32 ± 4.01 ^a^
BHT	/	1	90.56 ± 1.73 ^b^	79.38 ± 2.03 ^b^	14.39 ± 2.46 ^b^

Different letters (a and b) in the same column indicate significant differences (*p* < 0.05).

## Data Availability

The original contributions presented in this study are included in the article/Supplementary material, further inquiries can be directed to the corresponding authors.

## Supplementary Materials

The following supporting information can be downloaded at: https://www.mdpi.com/article/10.3390/molecules27217151/s1, Table S1: Primer information for PCR.

## Sample Availability

Samples of the GLHP are available from the authors.

## Abbreviations

CTRL: control; EtOH: alcohol; EtOH + GLHP, sample group; GLHP, peptide from goose liver hydrolysate; GSH-Px, glutathione peroxidase; *HO-1*, haem oxygenase 1; IEC, ion exchange chromatography; *NrF2*, nuclear factor E2; PBS, phosphate-buffered saline; ROS, reactive oxygen species; RP-LC, reversed-phase liquid chromatography; SEC, size exclusion chromatography; SOD, superoxide dismutase.
