# Supplementary material for: Isolation and Identification of Antioxidative Peptide from Goose Liver Hydrolysate to Ameliorate Alcohol-Mediated Oxidative Stress Damage in HHL-5 Hepatocytes
Source: Molecules. 2022 Oct 22;27(21):7151. doi: 10.3390/molecules27217151 (PMC9655001; doi:10.3390/molecules27217151)
Supplement: Supplementary file 1 [file molecules-27-07151-s001.zip › molecules-1951848-supplementary.pdf]

Supplementary Materials:

Table S1. Primers information for PCR

Gene	Forward (5'-3')	Reverse (5'-3')
GAPDH	ATTCAACGGCACAGTCAAGG	GCAGAAGGGGCGGAGATGA
NRF2	AGATGACCATGAGTCGCTTGC	CCAGCGAGGAGATCGATGAG
AhR	CCATCCCCGCTGAAGGAATTA	CTGCCAGTCTCTGATTTGTGC
NQO1	CATTGCAGTGGTTTGGGGTG	TCTGGAAAGGACCGTTGTCG
HO-1	AAGCCGAGAATGCTGAGTTCA	GCCGTGTAGATATGGTACAAGGA
